# Differential Impact of Pharmacokinetic and Pharmacodynamic Variability on Response to Combination Therapy

**DOI:** 10.1002/prp2.70281

**Published:** 2026-06-15

**Authors:** Kuteesa R. Bisaso, Ronald Kadada Karyaburo, Jackson K. Mukonzo, Ene I. Ette

**Affiliations:** ^1^ Breakthrough Analytics Limited Kampala Uganda; ^2^ Department of Pharmacology Makerere University College of Health Sciences Kampala Uganda; ^3^ Anoixis Corporation Natick Massachusetts USA

**Keywords:** combination therapy, interindividual variability, monte Carlo simulation, pharmacodynamic, pharmacodynamic drug–drug interactions, pharmacokinetic, response variability, signal‐reaction–stimulus–response model, sobol sensitivity analysis, system‐level variability

## Abstract

Interindividual variability (IIV) in drug response complicates both fixed‐dose design and individualized therapy. Understanding how pharmacokinetic (PK) and pharmacodynamic (PD) processes jointly shape this variability is essential, particularly in combination therapy, where multiple interacting pathways influence outcomes. This study employed a unified PK–Signal–Reaction–Stimulus–Response (PK–SRSR) modeling framework to quantify and compare the impact of IIV in PK and PD parameters under monotherapy and combination therapy. Monte Carlo simulations were used to generate response distributions across hypothetical physiological conditions, while Sobol variance‐based sensitivity analysis decomposed total response variability into contributions from individual parameters (FOI) and their interactions (TOI). Results showed that PD variability, especially that related to system‐level modulatory processes (S0A, S0B, βA, βB, and βiA), exerted a greater influence on overall response than drug effect level PD (SmaxA, SmaxB, KA, and KB) variability and PK (CLA/F and CLB/F) variability. Variability in response was most sensitive to S0B, βB (FOI > 34%, TOI > 32%) during monotherapy and SmaxA (FOI > 25%, TOI > 27%), SmaxB (FOI > 17%, TOI > 24%), and βB (FOI > 32%, TOI > 34%) during combination therapy. At 45% CV in all parameters, variability in response was lower during combination therapy (CV = 44%, 42%, and 33%) than during monotherapy (CV = 59%, 52%, and 53%) for additive, antagonistic, and synergistic PD interactions, respectively. Introducing a second drug targeting a modulatory pathway reduced response variability by stabilizing regulatory mechanisms, explaining previous observations in which patients achieve therapeutic response despite suboptimal plasma concentrations during combination therapy. Coordinated dose optimization of all agents, supported by response‐guided, system‐aware strategies, may yield more robust, consistent, and individualized treatment outcomes than traditional PK‐guided dosing approaches.

## 1 Introduction

Understanding interindividual variability (IIV) in drug response is fundamental to precision pharmacotherapy and efficient drug development. The overall response to a drug is governed by both pharmacokinetic (PK) and pharmacodynamic (PD) processes, each varying among individuals. These independent sources of variability interact nonlinearly as drug dose translates through PK and PD to clinical response. Because PD effects lie downstream of PK, variability in PD tends to have a greater influence on the final response [1]. Empirical evidence shows that interindividual variability in PD parameters often exceeds that in PK by a substantial margin, yet it remains less studied and is rarely incorporated into dose optimization [2]. This oversight undermines the effectiveness of target‐concentration–based dosing strategies and highlights the need to better characterize PD variability.

Pharmacokinetic–pharmacodynamic (PK/PD) modeling provides the principal computational framework for quantifying, understanding, and predicting the relationship between drug exposure, system variability, and clinical response. Mechanistic population models estimate IIV in key PK and PD parameters, identify predictable covariate effects, and separate PK from PD contributions to response variability [3]. However, simplified empirical PD models (e.g., Emax or sigmoidal functions) often leave the variability from target binding, signal transduction, and feedback regulation unexplained within residual random effects [4]. More mechanistic approaches, such as physiologically based pharmacokinetic (PBPK) and quantitative systems pharmacology (QSP) modeling, can capture pathway‐level variability and its impact on response, but their complexity limits parameter estimation and interpretability, necessitating simplified approaches [5, 6]. The signal‐reaction‐stimulus–response (SRSR) framework, which integrates both drug and biological system sub‐processes, may offer a practical balance [7]. By explicitly quantifying drug–receptor signaling and abridged downstream transduction PD sub‐processes under both monotherapy and combination therapy, it can link variability in specific PD sub‐processes to overall response variability [8].

Combination therapy, which involves the concurrent administration of multiple drugs and relies on pharmacodynamic drug–drug interactions (PDDI), influences PD variability more than PK variability. This occurs because combination regimens are selected to avoid PK interactions but perturb multiple biological pathways and molecular targets whose interactions are inherently patient‐specific. The resulting change in variability of the overall response may depend on whether the drug mechanisms are distinct or overlapping. The magnitude of these changes, however, remains poorly defined. Moreover, while the influence of PK and PD variability on monotherapy response has been investigated [1, 9], similar investigations for drug combinations, central to managing most chronic infectious and non‐communicable diseases, are lacking. The relative contributions and ranking of the PK and PD sub‐processes to the target response in such settings remain to be quantitatively established.

This sensitivity analysis and simulation study aimed to investigate the contributions of interindividual variability (IIV) in pharmacokinetic (PK) and pharmacodynamic (PD) drug‐ and system‐level sub‐processes to overall response variability, and to assess how the nature of pharmacodynamic drug–drug interactions (PDDIs) in combination therapy modulates these contributions.

## 2 Materials and Method

### 2.1 Exposure‐Response Modeling Framework

In this study, simulations of monotherapy and combination therapy were used to evaluate the impact of random variability in PK and PD on the variability in target response. Two hypothetical drugs A and B of similar molecular weight, with similar apparent clearance (CL/F) and dosing frequency (τ), were used to generate steady state concentration according to the exposure model in (Equation 1).

(1) $$\mathrm{Css}=\text{Dose}*F/\left(\mathrm{CL}*\tau \right).$$

The signal‐reaction‐stimulus–response (SRSR) model, previously called the stimulus–response model, was used to link exposure to response [7]. The target response (E) is given by the following set of equations:

(2) $$S_{A}=S0_{A}+\frac{{\text{Smax}}_{A}*\left[{\mathrm{Css}}_{A}\right]}{\left[{\mathrm{Css}}_{A}\right]+K_{A}},S_{B}=S0_{B}+\frac{{\text{Smax}}_{B}*\left[{\mathrm{Css}}_{B}\right]}{\left[{\mathrm{Css}}_{B}\right]+K_{B}}$$

(3) $$E=\frac{\left[\text{Emax}*S_{B}/\left(\beta_{B}+S_{B}\right)\right]*S_{A}}{\left[\left(\beta_{\mathrm{AB}}+\beta_{A}S_{B}\right)/\left(\beta_{B}+S_{B}\right)\right]+S_{A}}$$

where Emax is the maximal fractional response, Smax_A_ and Smax_B_ are respectively the maximal signal strength, operational efficacy, and transduction efficiency generated by the drugs A and B upon occupation of the receptor. K_A_ and K_B_ are, respectively the amounts of drug producing 50% of Smax_A_ and Smax_B_; S0_A_ and S0_B_ are the baseline stimulus due to endogenous substances sharing biochemical pathways affected by drugs A and B, respectively; β_A_ and β_B_ are the apparent potencies of stimuli due to biochemical pathways affected by drugs A and B, respectively, and β_AB_ is the square of the potency of the combination. The parameter β_AB_ was assumed to be directly proportional to one of the interacting signals' potency and thus not estimated directly but derived from β_AB_ = βi_A_*β_B_. where βi_A_ (the proportionality coefficient) was instead the estimated parameter as constrained by random sequential multi‐substrate enzyme kinetics [10]. The model parameters were used to calculate an interaction index INT as in (Equation 4) and determine the nature of PDDI using (Equation 5).

(4) $$\mathrm{INT}=\frac{\beta_{A}*\beta_{B}}{\beta_{\mathrm{AB}}}$$

(5) $$\text{Interaction}=\left\{\begin{array}{c} \text{Synergy},\mathrm{INT}<1\\ \text{Antagonism},\mathrm{INT}>1\\ \text{Additivity},\mathrm{INT}=1 \end{array}\right.$$

The parameters of the full PK–SRSR modeling framework were categorized into three groups, namely exposure parameters (CL_A_/F and CL_B_/F), drug‐level PD parameters (Smax_A_, Smax_B_, K_A_, and K_B_), and system‐level PD parameters (S0_A_, S0_B_, β_A_, β_B_, and βi_A_). The exposure parameters, drug effect parameters, as well as the baseline signals, system‐level PD parameters were assumed to be similar for both drugs. All parameters were selected on a normalized concentration scale (reference = 1) to span realistic pharmacodynamic orders of magnitude while enabling efficient exploration of weak, moderate, and strong signaling regimes without exhaustive parameter sampling, as shown in Table 1. The parameters were drawn from a simplified parameter set (0.01, 0.1, 1,10) to ensure numerical stability, computational efficiency, and interpretability. Within this framework, high‐affinity binding (K_A_ = K_B_ = 0.01), moderate signal transduction (SmaxA = SmaxB = 1), and a tenfold difference in pathway sensitivity (βA, βB = 0.1–1) reflect biologically plausible variation in receptor binding, signaling amplification, and enzyme kinetics. The potency of the combination (β_AB_) was assigned a uniform value for all experiments (0.1), and the interaction was assumed to be asymmetric in favor of A. The values of β_A_, βi_A_, and β_B_ were each assigned a value of 1 or 0.1 and combined in such a way as to create additive, synergistic, or antagonistic PDDI as per (Equations 4 and 5), allowing examination of how interaction type influences the propagation of variability.

**TABLE 1:** Pharmacokinetic and pharmacodynamic model parameters.

Parameter category	Parameter	Additivity	Antagonism	Synergy
**Exposure**	CL/F of A (L/h)	10	10	10
	CL/F of B (L/h)	10	10	10
	τ (hrs)	24	24	24
**Drug effect**				
	Smax_A_	1	1	1
	Smax_b_	1	1	1
	K_A_ (mg/L)	0.01	0.01	0.01
	K_B_ (mg/L)	0.01	0.01	0.01
**System**				
	S0_A_	0.1	0.1	0.1
	S0_B_	0.1	0.1	0.1
	β_A_	0.1	1	0.1
	β_B_	1	1	0.1
	β_iA_	0.1	0.1	1
	β_AB_	0.1	0.1	0.1
**Other**				
	INT	1	10	0.1
	Emax	1	1	1

### 2.2 Sensitivity Analysis and Simulation Experiments

Variance‐based (Sobol) sensitivity analysis was undertaken to assess the sensitivity of response to variability in the parameters of the full PK–SRSR modeling framework [11, 12]. The sensitivity analysis was implemented for both monotherapy (10 mg of A) and combo therapy (10 mg of both A and B) at a moderate IIV in parameters of 45% for the sample size of 1 000. The first‐order (FOI) and total‐order (TOI) indices were calculated for all parameters using the soboltouati method in version 1.30.1 of the Sensitivity package in R software [13, 14]. This enabled the identification and ranking of the parameters and sub‐processes most responsible for variability in overall response, both in monotherapy and combination therapy scenarios. The first‐order Sobol index (Sᵢ) measures the proportion of output variance attributable to variation in a single parameter alone, reflecting its direct effect on model behavior. The total‐order Sobol index (Sᵀᵢ) represents the overall influence of that parameter, including both its direct effect and interactions with other parameters. The difference between these indices, therefore, indicates the contribution of parameter interactions to output variability. As a global sensitivity method, Sobol analysis samples parameters across their full plausible ranges, enabling detection of nonlinear and interaction effects that local sensitivity methods cannot capture [11, 12].

In addition to the sensitivity analysis, a simulation study was undertaken to explore a dose range for the main drug A. In the monotherapy simulations, each of 0, 10, 20, 40, 80, 100, 200 mg of drug A was assigned to 50 individuals, while in the combination therapy dataset, all 350 individuals additionally received 10 mg of drug B. For each of the three categories of parameters, the log‐normal distribution was assumed and the interindividual variability (IIV) was varied between none (coefficient of variation = 0), low (coefficient of variation = 10%, 25% and 30%), moderate/normal (coefficient of variation = 45%), or high (coefficient of variation = 60%) while variability of the other two parameter categories was kept at 0%. The classification of interindividual variability as low, moderate, or high was based on conventional reported ranges [15]. The exposure‐response model was used to simulate steady‐state concentration (Css) and response (E). Each simulation was repeated 200 times to determine the sensitivity of the variability in Css and E to the IIV in the parameter categories. Each set of simulations was implemented under the assumption of additive, antagonistic, or synergistic system pathway interaction for both monotherapy with drug A and combination therapy with both drugs A and B. The simulations were implemented in NONMEM version 7.4. using the Perl‐speaks‐NONMEM tool kit version 3.6 [16, 17]. The generation of the simulation datasets, the pre and post‐simulation processing of the simulation results were implemented using R version 4.2.2 [14].

## 3 Results

### 3.1 Sensitivity Analysis

**FIGURE 1:**
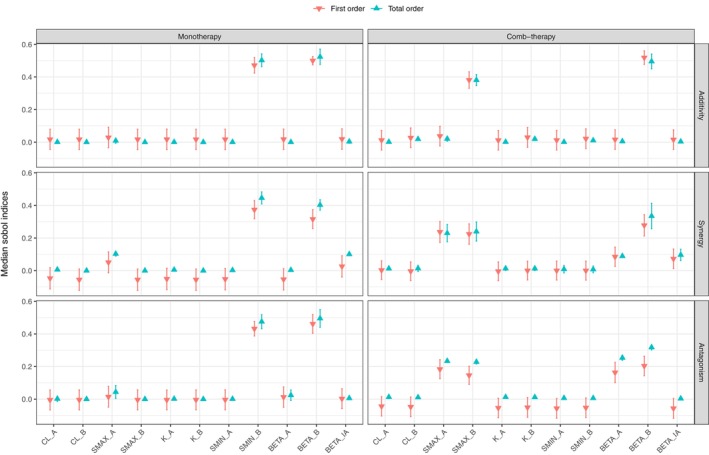
The first‐order and total‐order sensitivity indices of parameters under monotherapy and combination therapy.

During monotherapy with 10 mg of A, the response was more sensitive to system‐level PD parameters than to other parameters. Under additivity, the response was most sensitive to S0_B_, β_B_ (FOI > 49%, TOI > 49%), while the first and total‐order sensitivity indices for the other parameters were all less than 5%. Under synergy, the response was most sensitive to S0_B_, β_B_ (FOI > 34%, TOI > 32%), followed by Smax_A_, βi_A_ (FOI > 7%, TOI > 8%), and the rest of the parameters with sensitivity indices of less than 0.1%. Under antagonism, the response was most sensitive to S0_B_, β_B_ (FOI > 38%, TOI > 48%), while the first and total‐order sensitivity indices of the other parameters were all less than 0.5% (Figure 1).

However, when combined with 10 mg of drug B, the response was equally sensitive to both drug level and system‐level PD parameters. Under additivity, Smax_B_ (FOI = 38%, TOI = 40%) and β_B_ (FOI = 51%, TOI = 54%) were more significant than other parameters. Under synergy, Smax_A_ (FOI > 25%, TOI > 27%), Smax_B_ (FOI = 17%, TOI = 24%), and β_B_ (FOI = 32%, TOI = 34%) were more significant, and under antagonism Smax_A_ (FOI = 22%, TOI > 21%), Smax_B_ (FOI = 25%, TOI = 22%), β_A_ (FOI = 25%, TOI = 25%), β_B_ (FOI = 28%, TOI = 30%) were the more significant parameters (Figure 1).

### 3.2 Simulation Study

**FIGURE 2:**
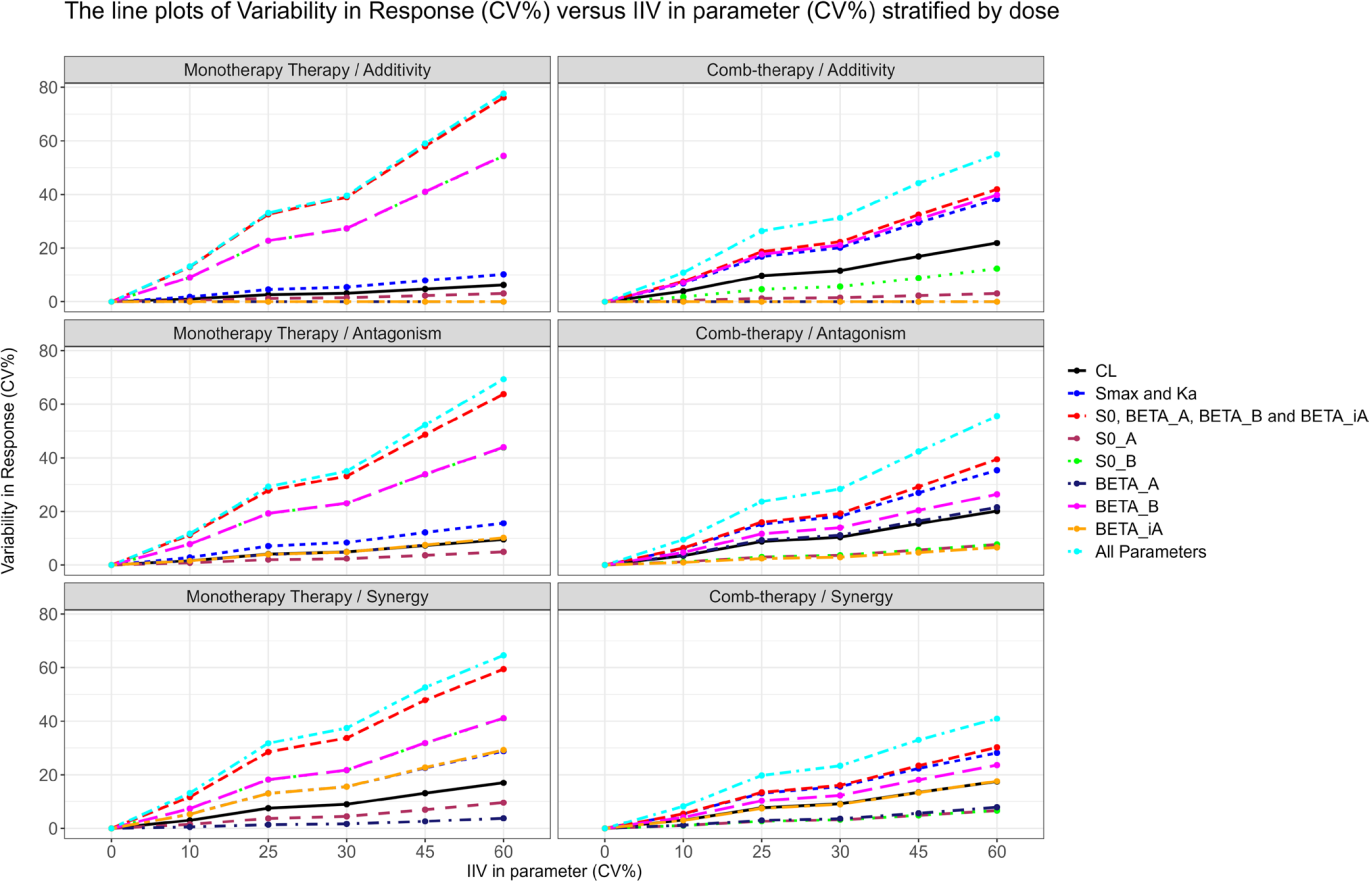
Effect of IIV in different PK and PD parameters on uncertainty in response under 10 mg of A and 0 mg of B (monotherapy) and 10 mg A and 10 mg of B (combination therapy).

Overall, response variability was lower under combination therapy than under monotherapy (Table 2). In both monotherapy and combination therapy, the variability in response increased with IIV in all parameters (Figure 2). The increase in variability in response under combination therapy was less than that under monotherapy (Figure 2). In all conditions, the increase in response variability was highest with system‐level PD parameters, followed by drug effect level parameters, and least with exposure parameters (Figure 2).

**TABLE 2:** Variability in response during monotherapy (10 mg of A) and combination therapy (10 mg of A and 10 mg of B) when the coefficient of variation in all parameters is 45%.

Nature of PDDI	Monotherapy (95% CI)	Combination therapy (95% CI)
Additivity	59.0 (54.4, 64.0)	44.2 (41.6, 47.0)
Antagonism	52.3 (48.2, 56.8)	42.4 (39.7, 45.4)
Synergy	52.6 (49.4, 56.1)	33.0 (31.0, 35.1)

Of the system‐level PD parameters, the increase was highest with S0_B_, β_B_ under monotherapy, and lowest with S0_A_, and β_A_. Moreover, the increase due to S0_A_, β_A_ was lower than that due to drug‐effect level PD parameters and exposure parameters. However, during combination therapy, the ranking under additivity was (β_B_ > Smax_A_, Smax_B_ K_A_, K_B_ > CL/F > S0_B_ > S0_A_ > β_A_ > βi_A_), and under synergy, it was Smax and K > β_B_ > β_A_ > CL/F > S0_A_ = S0_B_ > βi_A_. Under antagonism, it was Smax_A_, Smax_B_ K_A_, K_B_ > β_B_ > βi_A_ = CL/F > β_A_ > S0_A_ = S0_B_ (Figure 2).

**FIGURE 3:**
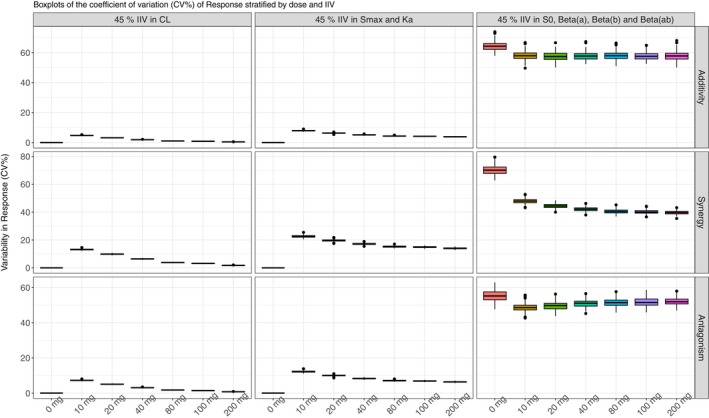
The dose variation of the influence of PK and PD parameters on uncertainty in response under monotherapy with A.

Increasing the dose of A from zero to 200 mg changes the contribution of each parameter category to the response in both monotherapy and combination therapy. During monotherapy, the influence of CL/F in the absence of the drug is zero, but the introduction of a low dose of A increases its influence, which then gradually falls back to zero at high doses. Similarly, the influence of drug effect level PD parameters is zero in the absence of drug but increases at a low dose of A and gradually reduces to a steady state level above zero (i.e., > 10% under synergy, and < 10% under additivity or antagonism) as the dose of A increases. The increase is highest under synergy and lowest under additivity. The influence of system‐level PD parameters is highest (> 50% increase in response at 45% IIV in parameters) in the absence of drug and sharply drops upon introduction of a low dose of A. It declines to a steady state value under synergy, but under additivity and antagonism, it gradually increases to a steady state as the dose of A increases. System‐level PD parameters have the largest influence on overall response, followed by drug‐level PD parameters and exposure parameters, respectively. The variation of parameter influence on response with dose of A under monotherapy is shown in Figure 3.

**FIGURE 4:**
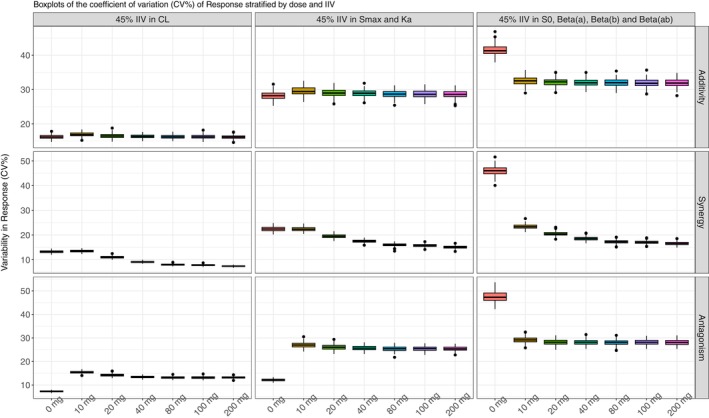
Effect of dose variation on the influence of PK and PD parameters on uncertainty in response under combination therapy of varying doses of A and 10 mg of B.

The variation of parameter influence on response with dose of A under combination therapy is shown in Figure 4. The influence of exposure and drug level PD parameters on the overall response under combination therapy is generally stronger than that during monotherapy, while the influence of system‐level PD parameters under combination therapy is generally lower than that under monotherapy. The trend in the influence of all parameters on response with dose is similar between monotherapy and combination therapy.

## 4 Discussion

Wide variability in drug response poses a major challenge to both fixed‐dose optimization and individualized therapy. Understanding and accounting for the underlying sources of variability is therefore essential for improving therapeutic outcomes. This simulation and sensitivity analysis study explored and ranked PK and PD contributors to response variability during monotherapy and combination therapy using a unified PK–SRSR framework.

Monte Carlo simulation was used to generate drug responses under diverse, hypothetical physiological and dosing conditions [18]. This approach enables isolation of PK, PD, and system‐level sub‐processes and examination of how interindividual variability (IIV) in each contributes to total response variability without the confounding effects of noisy real‐world data. Variance‐based global sensitivity analysis, using the Sobol method, was subsequently applied to quantify and rank the contribution of individual parameters and their interactions to overall variability. Using Monte Carlo or quasi‐random Sobol sampling, this robust, model‐independent approach is particularly valuable in nonlinear pharmacometric systems, where small parameter perturbations can produce large or counter‐intuitive changes in response.

The unified PK–SRSR framework ensured a consistent mechanistic representation of the IIV and PDDI processes that drive response variability across both monotherapy and combination therapy. The framework assumed steady‐state conditions, with equilibrium between plasma and effect‐site concentrations, and between receptor binding and downstream signal transduction processes. This avoided confounding due to temporal variation in response and allowed plasma levels to approximate target‐site exposure and pharmacodynamic response [19]. The SRSR model was adopted for the PD component because it integrates drug and system properties to describe receptor‐mediated signal generation, transduction, and interaction under both mono‐ and combination therapy. Drugs were assumed to act by mimicking, exaggerating, or interfering with endogenous ligand signaling [20]. Endogenous signaling pathways cross‐talk through enzyme‐mediated chemical reactions to produce a coordinated response [21, 22, 23]. Thus, drug interactions also occur via biochemical cross‐talk of the endogenous signaling pathways they stimulate [7, 8]. These enzyme‐mediated interactions were represented using multi‐substrate enzyme kinetics, enabling the model to capture biological interplay among signaling pathways [10]. Combination therapy was assumed to involve drugs with complementary, rather than identical, mechanisms of action [24].

Consistent with previous studies [2, 9, 25, 26], interindividual variability in both PK and PD processes contributed to overall variability in response. However, variability in PD was more influential than variability in PK, corroborating prior findings [1, 9, 26, 27, 28]. Among PD parameters, EC50, which reflects both drug and system properties such as receptor affinity, density, and signal amplification, was particularly influential [29, 30]. The proximity of the PD process to overall response has been postulated as the reason for its greater influence than PK on response. This highlights the limitations of PK‐guided dosing [31], especially in diseases where substantial pharmacodynamic variability exists, such as antiretroviral therapy, where immune responses to viral antigens influence treatment outcomes [32, 33].

Decomposition of the PD processes revealed that the response was more sensitive to system‐level processes than to drug‐specific properties. In the absence of treatment, baseline endogenous signals (S0_A_ and S0_B_) are responsible for the baseline response, and their IIV dominates response variability. During monotherapy with A, variability in the baseline modulating signal S0_B_ and its potency β_B_ were more influential than variability in the direct drug pathway (*S0*_*A*_, *β*_*A*_). This suggests that drug response is often more sensitive to biological modulators, such as feedback mechanisms and homeostatic processes, than to the drug's primary mechanism itself. These modulators are influenced by genetic [9], environmental, and psychological factors that biochemically induce placebo and nocebo effects [34] with contribute as high as 75% of the observed response [35]. Such findings emphasize the need to incorporate system‐level patient characteristics (e.g., genetic polymorphisms in signaling proteins, receptor density, and PD biomarkers) in precision therapy [36].

As the dose increased, the influence of baseline signals on response decreased, reflecting dose‐dependent stabilization of signaling pathways by the drug. Notably, when a second drug (B) was introduced, the influence of the modulating signal S0_B_ diminished and was superseded by drug effect parameters Smax_A_ and Smax_B_. Thus, introducing a second drug that targets a modulatory pathway can reduce interindividual variability by stabilizing the modulatory biological processes underlying PD variability. The second drug effectively standardizes signaling and feedback regulation, thereby diminishing differences in receptor sensitivity across patients, leading to more consistent therapeutic responses even when PK variability persists. This mechanism plausibly explains observations in which patients fail to reach target plasma concentrations of one drug in the combination yet still achieve the desired response [37, 38]. It also explains why target concentrations derived from monotherapy may not apply to combination therapy, where system‐level stabilization modifies the exposure–response relationship.

Relying on monotherapy‐based targets could thus lead to misestimation of effective dosing in combined regimens. Treatment success in combination therapy may depend more on network‐level PD interactions than on plasma drug concentrations, supporting a shift toward response‐guided dosing strategies over concentration‐based approaches. However, if PK‐guided optimization is to be undertaken in multi‐drug regimens, joint optimization of all agents is preferable to single‐agent adjustment, as pharmacodynamic interactions alter sensitivity to PK and PD variability.

Finally, while the PK–SRSR framework provided valuable mechanistic insights, several limitations apply. The analysis relied on assumed model structures, steady‐state simplifications, and hypothetical parameter distributions, which may not fully capture biological complexity or dynamic feedback. Assuming steady state emphasizes concentration‐dependent mechanisms and may underrepresent time‐dependent processes such as delayed signaling, feedback regulation, receptor trafficking, or adaptive adaptation, phenomena where identical concentrations produce divergent responses based on exposure history. and can alter parameter influence during treatment. Consequently, the estimated sensitivity rankings reflect variability under quasi‐equilibrium conditions rather than the full temporal evolution of drug response. Likewise, modeling parameters as log‐normally distributed captures positive, multiplicative biological variability expected in multistep biochemical signaling cascades and restricts simulations to physiologically plausible parameter ranges but does not fully represent structural heterogeneity arising from distinct biological subpopulations or pathway rewiring. These assumptions, therefore, facilitate stable variance decomposition but may smooth certain sources of dynamic or biological variability. Thus, the results primarily identify potential mechanistic drivers of response variability within the assumed model structure rather than fully capturing the complexity of real clinical responses. Consequently, findings regarding variability dominance and combination stabilization represent mechanistic hypotheses about network architecture rather than quantitative clinical predictions, requiring dynamic validation to confirm temporal generalizability.

In this study, signaling pathways are discussed in general terms as PD processes that occur between receptor occupancy and observed response. Specific signaling pathways would require more detailed quantitative systems pharmacology modeling, which was beyond the scope of this study. The absence of empirical validation and biological noise further limits clinical extrapolation. Therefore, experimental and clinical studies are required to confirm and refine these simulation‐based findings before translation into dosing practice.

## 5 Conclusion

This study demonstrates that pharmacodynamic processes and their modulation play a dominant role in determining interindividual variability in drug response. In combination therapy, pharmacodynamic interactions can stabilize biological responses, reducing variability and allowing therapeutic success even at sub‐target concentrations. Consequently, joint optimization of all drugs in a combination regimen is preferable to PK‐guided adjustment of a single agent. The findings emphasize that monotherapy‐based concentration targets may not be suitable for optimizing dosing in multi‐drug contexts, where pharmacodynamic interactions reshape system sensitivity. The combined use of Monte Carlo simulation and Sobol sensitivity analysis provides a robust quantitative framework for understanding and predicting how variability in PK, PD, and system parameters translates into variability in therapeutic response. These mechanistic and methodological insights support the development of response‐guided, rather than exposure‐guided, dosing strategies for complex therapies involving interacting drugs.

## Author Contributions

K.R.B. conceived, planned the study, analyzed the data, interpreted the results, and wrote the manuscript. R.K.K. performed the simulation and sensitivity analysis. J.K.M. reviewed and edited the manuscript. E.I.E. reviewed and edited the manuscript and analysis.

## Conflicts of Interest

The authors declare no conflicts of interest.

## Supporting information


**Figure S1:** Schematic representation of the Signal‐Reaction‐Stimulus–Response framework.

## Data Availability

Data sharing not applicable to this article as no real world datasets were generated or analyzed during the current study.

## Appendix A

Derivation of the Signal–Reaction–Stimulus–Response (SRSR) Framework and Its Application to Selected Combination Therapies.

### 1 Conceptual basis of the SRSR framework

The SRSR framework conceptualizes drug action as a sequence in which drug–target interactions generate biochemical signals, which interact through biochemical reactions, produce an integrated stimulus, and ultimately lead to a measurable physiological response. This structure is particularly useful for understanding combination therapies, where signals generated by different drugs interact within biological networks before producing the final pharmacodynamic outcome. The framework helps explain how drugs with complementary mechanisms can stabilize biological systems, amplify therapeutic effects, and reduce response variability. The framework, therefore, integrates ideas from receptor theory, the operational model of agonism, multisubstrate enzyme kinetics, and stimulus–response transduction models into a single structure applicable to both monotherapy and combination therapy. At a high level, the framework can be written as:

Drug concentration → Target binding → Signal generation → Signal integration (reaction) → Stimulus → Response

But the focus is on: Signal generation → Signal integration → Stimulus → Response

Following the guidelines set out by Reid et al. [24], this analysis assumes pharmacokinetic interactions between combination components are minimized, allowing focus on pharmacodynamic interactions at the signal integration layer.

To derive this structure formally, each component is introduced sequentially.

### 2 Receptor binding as the origin of the drug‐generated signal

The first stage is drug–receptor binding. For a drug A interacting reversibly with a receptor R:

A+R⇌AR,

with the equilibrium dissociation constant given as follows:

$$\frac{\left[A\right]\left[R\right]}{\left[\mathrm{AR}\right]}=K_{d},$$

and the total receptor concentration

$$R_{T}=\left[R\right]+\left[\mathrm{AR}\right]$$

Solving for receptor occupancy gives:

$$\left[\mathrm{AR}\right]=\frac{\left[A\right]R_{T}}{K_{d}+\left[A\right]}.$$

This is the classical occupancy relation. By itself, it only describes how much of the drug is bound, not how much effect is produced. The SRSR framework interprets receptor occupancy as the source of a biochemical signal. If signal generation is proportional to active complex formation, then the drug‐generated signal may be written as

$$S_{A}=\alpha_{A}R_{T}\left[\mathrm{AR}\right]$$

or, after substituting the occupancy expression,

$$S_{A}=\alpha_{A}R_{T}\frac{\left[A\right]}{K_{d,A}+\left[A\right]}$$

Here, α_A_ is a signal generation efficiency term converting receptor occupancy into an intracellular signal. Thus, receptor binding is reinterpreted as signal generation, not yet response.

For a second, drug B

$$S_{B}=\alpha_{B}R_{T}\frac{\left[B\right]}{K_{d,B}+\left[B\right]}$$

In the presence of endogenous ligand‐generated baseline signals, one may also define baseline signal terms S_0A_, S_0B_, etc., representing physiological signaling in the absence of a drug.

### 3 Operational model as the link from occupancy to effective stimulus

The classical occupancy model cannot explain why drugs with similar occupancy may have different efficacy. For this, the Black–Leff operational model is introduced [39]. In the operational model, the effect of a drug depends not only on affinity but also on efficacy and transduction efficiency. For the agonist A:

$$E=\frac{E_{m}\tau \left[A\right]}{K_{d,A}+\left[A\right]+\tau \left[A\right]}$$

where:

E_m_ is the maximal system response.

K_d_ is affinity.

τ is the transducer ratio reflecting efficacy, receptor density, and coupling efficiency.

This can be recast in signal language. Define the effective signal generated by drug A as follows:

$$S_{A}=\tau_{A}\frac{\left[A\right]}{K_{d,A}+\left[A\right]}$$

Then the response emerges from a transduction function.

$$E=\frac{E_{m}S_{A}}{1+S_{A}}$$

This step is important because it shows that the pharmacodynamic driver is not concentration alone, but a composite signal incorporating drug affinity, efficacy, receptor density, and transduction efficiency. It also clarifies why parameters such as EC_50_ are hybrid quantities: They reflect both drug properties and system properties.

Within the SRSR framework, this operational step becomes the bridge between receptor occupancy and biologically meaningful signal strength.

### 4 Cleland multisubstrate model as the basis for signal integration

Drugs rarely act in isolation from endogenous pathways or other drugs. To represent the interaction of multiple signals, the SRSR framework uses Cleland multisubstrate enzyme kinetics. In this view, intracellular signal integration is treated analogously to a multisubstrate enzyme reaction, where signals are the effective inputs to a biochemical node.

For a two‐signal system, if signals S_A_ and S_B_ converge on an integration node, the velocity of stimulus generation may be written in generic form as follows:

$$v\left(S_{A},S_{B}\right)=\frac{V_{\max}S_{A}S_{B}}{\beta_{\mathrm{AB}}+\beta_{A}S_{B}+\beta_{B}S_{A}+S_{A}S_{B}}$$

where β_A_ and β_B_ are the apparent potencies of signals, S_A_ and S_B_, and β_AB_ is the square of the potency of the combination, βi_A_ is the dissociation constant of S_A_, the first substrate to add during sequential reactions. Assuming rapid equilibrium, βi_A_*β_B_ = βi_B_*β_A_[7]. More generally, the exact denominator depends on whether the integration node behaves as a sequential, random (β_AB_ = β_A_*β_B_), sequential ordered (β_AB_ = βi_A_*β_B_), or ping‐pong (β_AB_ = 0) mechanism, but the key point is that both signals contribute jointly to the rate of integrated stimulus formation. In standard bi‐substrate kinetics, it is often assumed that only the ternary complex (E_nz_S_A_S_B_) is productive. However, in many biological systems, such as those involving partial inhibition or non‐essential activation, the binary complexes (E_nz_S_A_) and (E_nz_S_B_) are catalytically active and contribute to the total reaction velocity. E_nz_ is the enzyme. Under such a flexible framework, the velocity of stimulus generation may be written in generic form as

$$v\left(S_{A},S_{B}\right)=\frac{V_{\max,A}\beta_{B}S_{A}+V_{\max,B}\beta_{A}S_{B}+V_{\max,\mathrm{AB}}S_{A}S_{B}}{\beta_{\mathrm{AB}}+\beta_{A}S_{B}+\beta_{B}S_{A}+S_{A}S_{B}}$$

This is the essential move of the SRSR model: Drug‐generated and endogenous signals are treated as biochemical inputs that interact at reaction nodes according to multisubstrate kinetics. Under monotherapy, one drug signal interacts with endogenous signals. Under combination therapy, two or more drug signals join the endogenous signals network. Thus, if signal S_A_ is the direct signal from drug A and S_0B_ is an endogenous cross‐talking signal, then the integrated node output may be

$$v=v\left(S_{A},S_{0B}\right)$$

If drug B is introduced and substitutes for or modulates that endogenous pathway, then the node output becomes

$$v=v\left(S_{A},S_{0B}\right)$$

or, when baseline and drug‐modified signals coexist

$$v=v\left(S_{A},S_{0B}+S_{B}\right)$$

This gives the framework a mechanistic way to describe synergy, additivity, antagonism, and buffering of variability.

$$\mathrm{INT}=\frac{\beta_{A}*\beta_{B}}{\beta_{\mathrm{AB}}}$$

$$\text{Interaction}=\left\{\begin{array}{c} \text{Synergy},\mathrm{INT}<1\\ \text{Antagonism},\mathrm{INT}>1\\ \text{Additivity},\mathrm{INT}=1 \end{array}\right.$$

In enzyme kinetics, INT < 1 favors ternary complex formation, whereas INT > 1 disfavors it.

However, at the point of signal integration, the SRSR framework can be extended to accommodate other mechanisms of convergence. These mechanisms can be classified into type A or type B. Type A integration occurs through shared biochemical nodes (particularly multisubstrate enzymatic reactions) while type B occurs through physiological feedback systems. Type A combinations permit quantitative kinetic formalism following mass action laws, while type B combinations are better described by physiological control theory.

### 5 Dynamic stimulus formation

The output of the integration node is not itself the final effect. It is the stimulus that drives the response. In dynamic form, stimulus may accumulate or decay over time:

$$\frac{dX^{*}}{\mathrm{dt}}=k_{\text{in}}v\left(S_{A},S_{B},\ldots \right)-k_{\text{out}}X^{*}$$

Here, X* is the integrated stimulus output from the integration node.

K_in_ converts node activity to stimulus formation.

K_out_ represents stimulus dissipation, turnover, or homeostatic counter‐regulation.

At steady state,

$$X^{*}=\frac{k_{\text{in}}}{k_{\text{out}}}v\left(S_{A},S_{B},\ldots \right)$$

This is the stimulus quantity that enters the response model. The dynamic representation is important because it allows onset delays, persistence, and turnover effects to be incorporated. Even when steady‐state conditions are assumed, this equation clarifies that the stimulus is the result of ongoing signal integration and removal.

### 6 Direct and saturating response generation

The final stage of the SRSR framework is the conversion of stimulus into a measurable response. Two cases are useful. If the node output or stimulus itself is directly observable, the response may be written as follows:

$$E=E_{0}+c\;\;X^{*}$$

or, if saturation is expected

$$E=E_{0}+\frac{E_{m}\;X^{*}}{K+X^{*}}$$

E_0_ captures contributions to the measured response that do not originate from the modeled signaling mechanism, including measurement offset/error, parallel biological processes, or unmodeled pathways.

This is appropriate when the measured outcome is closely linked to the integrated biochemical node.

This is the formal derivation of the SRSR framework: Receptor binding creates signals; operational efficacy transforms occupancy into effective signaling strength; multisubstrate kinetics govern signal integration; dynamic turnover forms stimulus; and direct or transduced pathways convert stimulus into response.

In the most simplified direct response form as used in the current analysis, E0 is assumed to be zero, and E_max_ = c*k_in_*V_max_/k_out_.

**Drug Classes to which the SRSR Framework Applies**

The signal–stimulus–response (SRSR) framework is broadly applicable to drug classes whose therapeutic effects arise from receptor‐mediated signaling or modulation of biochemical pathways that generate intracellular signals before producing a measurable physiological response. This includes receptor agonists and antagonists, such as β‐adrenergic agonists (e.g., salbutamol), opioids (e.g., morphine), corticosteroids (e.g., prednisone), and dopamine receptor antagonists (e.g., haloperidol), where receptor occupancy initiates signaling cascades that determine downstream physiological effects. The model also applies to enzyme inhibitors and activators, including kinase inhibitors targeting signaling pathways (e.g., imatinib targeting BCR‐ABL or PI3K–AKT–mTOR pathway inhibitors), phosphodiesterase inhibitors (e.g., sildenafil), and protease inhibitors (e.g., ritonavir), whose actions alter enzymatic reactions governing cellular signaling networks. In addition, the SRSR framework is suitable for signaling pathway modulators and immunomodulatory agents, such as cytokine modulators (e.g., tocilizumab) and targeted anticancer therapies that influence intracellular signaling cross‐talk. The framework is particularly useful for combination therapies with complementary mechanisms, where drug‐generated signals interact through shared biological pathways. Examples include tenofovir–lamivudine–efavirenz in antiretroviral therapy and oncology combinations such as dabrafenib–trametinib targeting BRAF–MEK signaling. In such regimens, the therapeutic response emerges from the integration of drug‐induced and endogenous signals within interconnected biochemical pathways.

**Application to Selected Combination Therapies**

### 7 Tenofovir–lamivudine–efavirenz in antiretroviral therapy

The tenofovir–lamivudine–efavirenz regimen is a simplified example of SRSR applied to the inhibition of a viral enzymatic pathway. Tenofovir and lamivudine are intracellularly activated nucleos(t)ide analogues that generate inhibitory signals by competing with endogenous nucleotides during reverse transcription and terminating viral DNA chain elongation. Efavirenz binds allosterically to HIV reverse transcriptase, generating a distinct inhibitory signal that reduces enzyme catalytic efficiency.

In SRSR terms, the three drugs generate separate signals at the target level. These signals converge at the reverse transcriptase reaction node, which is the multisubstrate integration point. Here, the integration node is also the target itself (reverse transcriptase). The integrated stimulus is suppression of viral DNA synthesis, and the final response is a reduction in viral replication and viral load. Because NRTIs and NNRTIs inhibit the same enzyme through different mechanisms (competitive/substrate incorporation vs. non‐competitive/allosteric), this combination exemplifies the Reid criterion of complementary mechanisms at a single enzymatic target. The combined stimulus is stronger and more stable than that produced by any single drug, and variability in one component can be buffered by the others. This is a simpler case: Direct enzyme targeting rather than pathway convergence.

### 8 Dabrafenib–trametinib targeting BRAF–MEK signaling

The dabrafenib–trametinib combination provides a canonical example of SRSR in a signaling cascade. Dabrafenib inhibits mutant BRAF kinase, generating a signal that reduces phosphorylation of MEK. Trametinib inhibits MEK1/2 directly, generating a second signal that blocks activation of ERK. Here, the reaction layer corresponds to the MAPK cascade, where sequential kinase reactions transmit proliferative signals. The two drugs act at adjacent nodes, and their signals converge on the downstream output of the pathway, namely ERK signaling. The integrated stimulus is suppression of the MAPK pathway output, and the final response is reduced tumor cell proliferation and tumor burden. The combination is especially effective because inhibition at the MEK level suppresses feedback‐driven reactivation that can occur with BRAF inhibition alone.

### 9 General implication

Across all four examples, the SRSR framework shows that combination therapy is best understood not as a simple addition of drug effects but as integration of biochemical signals within biological networks. In antiretroviral therapy, signals converge on a viral enzyme. In antihypertensive therapy, they converge on physiological determinants of arterial pressure. In oncology, they converge on pathway signaling output. In each case, the framework captures how complementary drug actions can suppress endogenous compensation, stabilize system behavior, and reduce sensitivity of the final response to variability in any single component.
