# Supplementary figures and images for: Differential Impact of Pharmacokinetic and Pharmacodynamic Variability on Response to Combination Therapy
Source: Pharmacol Res Perspect. 2026 Jun 15;14(4):e70281. doi: 10.1002/prp2.70281 (PMC13269674; doi:10.1002/prp2.70281)

Figure S1: Schematic representation of the Signal-Reaction-Stimulus-Response framework


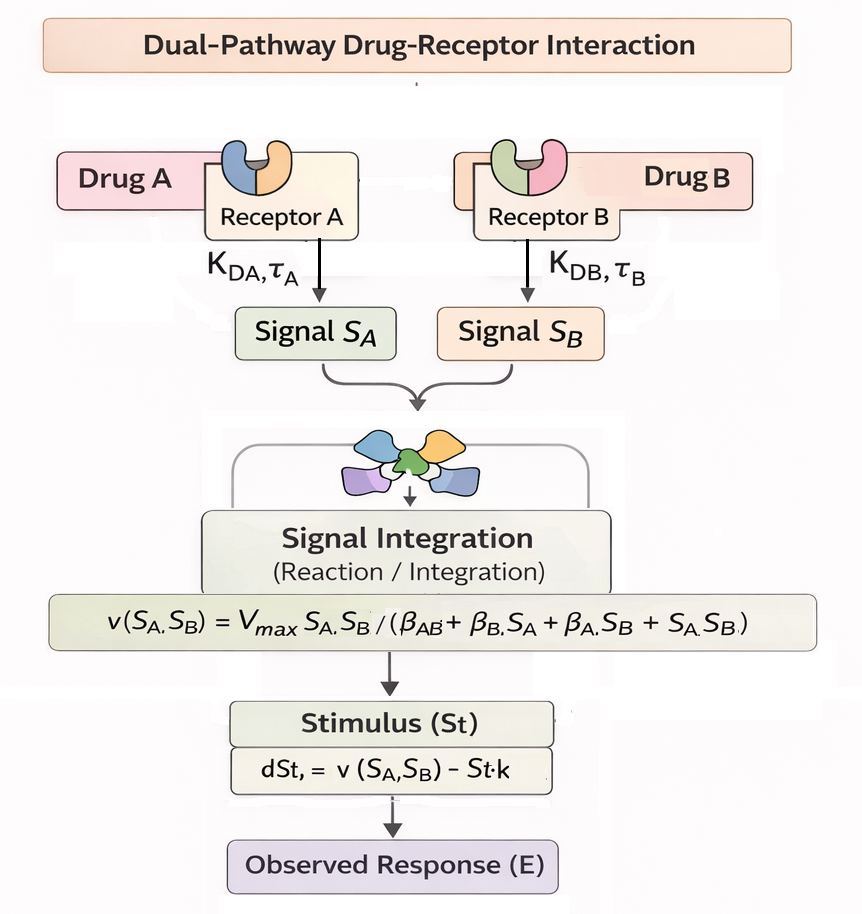

Supplement: Supplementary file 1 — Figure S1: Schematic representation of the Signal‐Reaction‐Stimulus–Response framework. [file PRP2-14-e70281-s001.docx]
